# Tuning the Structure of Nylon 6,6 Electrospun Bundles to Mimic the Mechanical Performance of Tendon Fascicles

**DOI:** 10.3389/fbioe.2021.626433

**Published:** 2021-04-06

**Authors:** Alberto Sensini, Michael H. Santare, Emily Eichenlaub, Ellen Bloom, Carlo Gotti, Andrea Zucchelli, Luca Cristofolini

**Affiliations:** ^1^Advanced Applications in Mechanical Engineering and Materials Technology – Interdepartmental Center for Industrial Research (CIRI-MAM), Alma Mater Studiorum-Università di Bologna, Bologna, Italy; ^2^Department of Mechanical Engineering, University of Delaware, Newark, DE, United States; ^3^Department of Biomedical Engineering, University of Delaware, Newark, DE, United States; ^4^Department of Industrial Engineering, Alma Mater Studiorum-Università di Bologna, Bologna, Italy; ^5^Health Sciences and Technologies – Interdepartmental Center for Industrial Research (CIRI-HST), Alma Mater Studiorum-Università di Bologna, Bologna, Italy

**Keywords:** electrospinning, nanofibers, nylon 6, 6 bundles, tendon fascicles, bioinspired structures, biomechanical modeling

## Abstract

Tendon and ligament injuries are triggered by mechanical loading, but the specific mechanisms are not yet clearly identified. It is well established however, that the inflection and transition points in tendon stress-strain curves represent thresholds that may signal the onset of irreversible fibrillar sliding. This phenomenon often results in a progressive macroscopic failure of these tissues. With the aim to simulate and replace tendons, electrospinning has been demonstrated to be a suitable technology to produce nanofibers similar to the collagen fibrils in a mat form. These nanofibrous mats can be easily assembled in higher hierarchical levels to reproduce the whole tissue structure. Despite the fact that several groups have developed electrospun tendon-inspired structures, an investigation of the inflection and transition point mechanics is missing. Comparing their behavior with that of the natural counterpart is important to adequately replicate their behavior at physiological strain levels. To fill this gap, in this work fascicle-inspired electrospun nylon 6,6 bundles were produced with different collector peripheral speeds (i.e., 19.7 m s^–1^; 13.7 m s^–1^; 7.9 m s^–1^), obtaining different patterns of nanofibers alignment. The scanning electron microcopy revealed a fibril-inspired structure of the nanofibers with an orientation at the higher speed similar to those in tendons and ligaments (T/L). A tensile mechanical characterization was carried out showing an elastic-brittle biomimetic behavior for the higher speed bundles with a progressively more ductile behavior at slower speeds. Moreover, for each sample category the transition and the inflection points were defined to study how these points can shift with the nanofiber arrangement and to compare their values with those of tendons. The results of this study will be of extreme interest for the material scientists working in the field, to model and improve the design of their electrospun structures and scaffolds and enable building a new generation of artificial tendons and ligaments.

## Introduction

Tendons and ligaments (T/L) have a complex and multiscale fibrous structure (Kastelic et al., 1978; Kannus, 2000; Goh et al., 2014). In particular, the nanometric collagen type I fibrils (diameter 50–250 nm) are the building blocks of this hierarchical arrangement (Kannus, 2000). Scaling up the T/L structure, bunches of collagen fibrils group together aligning their axis in the direction of the whole tissue longitudinal axis, producing the typical T/L fascicles (diameter range from few tens up to hundreds of micrometers) (Kastelic et al., 1978; Kannus, 2000; Murphy et al., 2016). The mechanical behavior of intact and injured T/L tissue is related to deformation on these various scales, but the exact mechanisms are still not completely clear.

Collagen fibers are the main providers of structural stiffness and strength in tendons and are predominately, but not perfectly, aligned in the axial, load-bearing direction of the tissue. This alignment explains the highly anisotropic properties of tendon tissues. Lynch et al. (2003) found the modulus of both the toe-region and linear region to be two orders of magnitude greater in the fiber-aligned direction and the Poisson’s ratio to be six times greater for loading in the direction of the fibers than transversely. However, some degree of off-axis alignment or crimping of these fibers explains the non-linearity seen in the early portions of their stress-strain curves. The toe-region of the stress-strain curve has low stiffness since the fibers are not perfectly aligned and the stiffness increases as the fibers become more aligned under load (Silver et al., 2000; Hansen et al., 2002; Weiss et al., 2002; Lake et al., 2009). The multiscale strain transfer is also dependent on the orientation of fibers. Han et al. (2013) observed strain heterogeneity within native tissue from the tissue to the cellular level. The strain transfer between the tissue level, micro-scale level and cellular level experience about 11–15% attenuation.

Lee et al. (2017) conducted quasi-static, *in vitro*, mechanical testing on rat tail tendon fascicles to investigate the multiscale mechanisms of damage. Each tendon fascicle was randomly assigned to an initial 2, 4, 6, or 8% strain. The subsequent deformation to failure was recorded under a confocal microscope while transition strain, inflection point strain and linear modulus at the tissue-level were measured. They concluded that the inflection point of the stress-strain curve corresponded to the damage threshold and that interfibrillar sliding was the micro-mechanism associated with that damage. This damage resulted in a decrease in stiffness and an elongation of the toe region in the stress-strain response of the tendon. Creep testing of human Achilles tendon showed that time to failure and applied stress were not correlated. However, time to failure was inversely correlated to the initial strain (Wren et al., 2001).

Effectively replacing the tendon structure when a severe injury occurs requires an improvement of the prosthetic devices and scaffolds currently on the market (Chen et al., 2014). Among the several techniques currently used to regenerate and replace (R/R) these tissues, electrospinning appears to be the most promising (Bosworth and Downes, 2011; Gomes et al., 2015). The key feature of this technology is its ability, using high electrostatic fields, to stretch polymeric solutions, thereby obtaining nanometric fibers similar to the collagen fibrils of the tendon’s extracellular matrix (Sensini and Cristofolini, 2018; Sensini et al., 2021). Moreover, by tuning the process parameters, the shape and structure of the electrospinning drum collector as well as its rotational speed, it is possible to obtain three-dimensional structures (i.e., bundles or yarns) similar to those in tendon fascicles (Ramakrishna et al., 2005; O’Connor and McGuinness, 2016). Electrospun bundles and yarns of axially aligned nanofibers have been shown to mimic the natural tissue mechanics as well as to promote cellular infiltration and extracellular matrix production (Bosworth et al., 2013; Domingues et al., 2016; Pauly et al., 2017; Sensini et al., 2017, 2018c). Focusing on the total replacement of the injured tissue, the electrospun nylon 6,6 has demonstrated a suitable structure and mechanics for this purpose (Sensini and Cristofolini, 2018, Sensini et al., 2019a). Moreover, nylon 6,6 is a widely-employed biomaterial in clinical applications, such as suture wires or implantable non-resorbable devices (Maitz, 2015; Krysiak et al., 2020). Past studies have focused on the average stiffness and failure properties of artificial tissue surrogates. In this study, we focus on the biomechanically-relevant stress-strain behavior in the physiological strain range corresponding to the toe region up to the inflection point.

## Materials and Methods

### Materials

Nylon 6,6 pellets, kindly provided by DuPont (Wilmington, DE, United States), were dissolved in a trifluoroacetic acid (TFA) (Carlo Erba, Milan, Italy) and acetone (AC) (Sigma Aldrich, St. Louis, MO, United States) mixture, obtaining the following solution: 15% (w/v) solution of Nylon 6,6 dissolved in TFA:AC = 50:50 (v/v).

### Electrospun Bundles Preparation

To electrospin nanofibers similar to the tendon collagen fibrils (mean diameter = 0.1–0.3 μm) (Kannus, 2000; Goh et al., 2014), a commercial electrospinning machine (Spinbow srl, Bologna, Italy) equipped with a linear sliding spinneret and a rotating drum collector (length = 405 mm, diameter = 150 mm) was used. To easily detach the nanofibers mats, the drum was covered with a sheet of polyethylene (PE) coated paper (Turconi S.p.A, Ceriano Laghetto, Italy). Four metallic needles (inner diameter = 0.84 mm) were fed, through PTFE tubes, by four syringes carrying the polymeric solution with a flow rate of 0.5 ml h^–1^ controlled by a syringe pump (KD Scientific 200 series, IL, United States). The needles where fixed on a sliding spinneret with a linear excursion of 100 mm, along the drum axis, and with a speed of 1,500 mm min^–1^. The needle-to-collector distance was set at 160 mm with an applied voltage of 20 kV. All the electrospinning sessions were carried out at a temperature of 21–23°C and a relative humidity of 20–30%.

**FIGURE 1:**
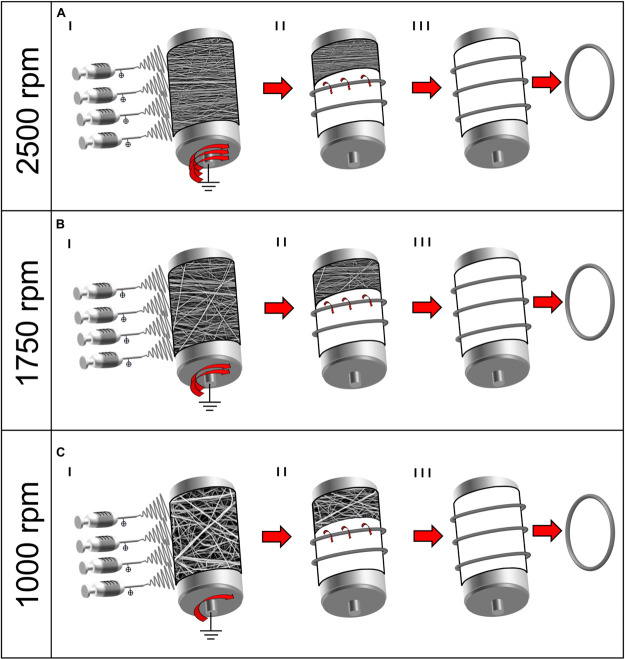
Schematic representation of the electrospinning procedure for the bundle production: **(A)** 2,500 rpm; **(B)** 1,750 rpm; and **(C)** 1,000 rpm. (I) Mats of nanofibers were electrospun on the rotating drum collector at different speeds. (II) The mats were cut in stripes and rolled on the drum and (III) pulled off the drum obtaining ring-shaped bundles.

To investigate the variations in nanofiber alignment and mechanical properties, a different drum peripheral speed and electrospinning time was set for each sample category: (A) 19.6 m s^–1^ (drum rotation = 2,500 rpm) for 3 h; (B) 13.7 m s^–1^ (1,750 rpm) for 2 h; (C) 7.9 m s^–1^ (1,000 rpm) for 2 h. At the end of the process, 6–8 μm thick nanofibrous mats were obtained (Figures 1AI–CI, 2A). The thickness of the mats was obtained by 72 measures along the mats’ length using a digital indicator [ALPA, Pontoglio (BS), Italy].

**FIGURE 2:**
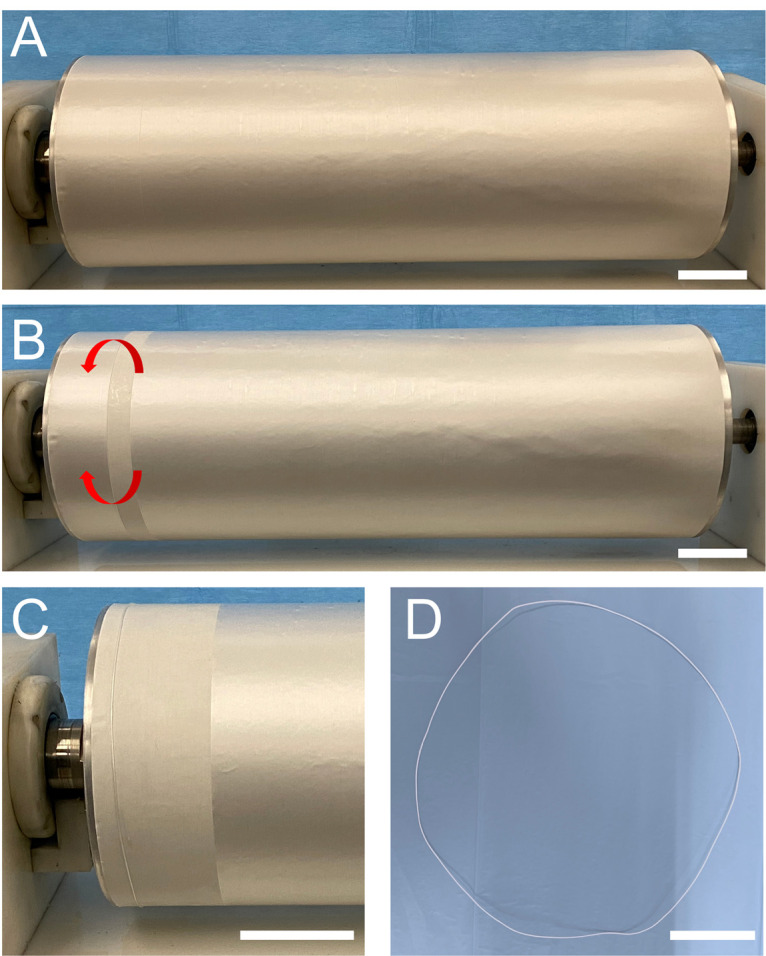
Series of images showing the procedure for a ring-shaped bundle preparation: **(A)** mat of nanofibers electrospun on the rotating drum collector; **(B)** a stripe of the mat that was wrapped up on the drum; **(C)** the ring-shaped bundle just wrapped on the drum is visible on the left, while the rest of the mat, ready for being wrapped, is still on the right; and **(D)** ring-shaped bundle pulled off the drum (scale bars = 50 mm).

In order to reproduce the tendon fascicles (mean diameter = 400–600 μm) (Kannus, 2000; Goh et al., 2014), bundles of nanofibers were prepared by following a previously reported procedure (Sensini et al., 2017, 2018b, 2019a,b). Briefly, the mats described above were cut in stripes following the circumference of the drum collector. The stripes were then manually rolled (Figures 1AII–CII, 2B,C) and pulled off from the drum, obtaining ring-shaped bundles (mean diameter = 450–650 μm) (Figures 1AIII–CIII, 2D).

### Morphological Characterization

The bundle surfaces were gold-sputtered to be investigated with a Scanning Electron Microscope at 10 kV (SEM, Phenom Pro-X, PhenomWorld, Eindhoven, Netherlands). The opensource software ImageJ (Liu, 1991) was used to measure the diameters of 200 nanofibers, for each bundles category. The nanofiber diameter distribution was investigated considering 200 measures from each bundle category (based on SEM images with a magnification of 15,000×). The bundle diameters were measured with an optical microscope (Axioskop, Zeiss, Pleasanton, CA, United States) equipped with a camera (AxioCam MRc, Zeiss, Pleasanton, CA, United States) and are reported as mean and standard deviation of 20 measures. We evaluated the nanofiber orientation with the Directionality plugin of ImageJ (Schindelin et al., 2012; Schneider et al., 2012). This approach allowed us to quantify the number of nanofibers within a given angle range from the axis, using a Local Gradient Orientation method, following a previously validated procedure (Sensini et al., 2018a). For each sample category the analysis was performed on five images (magnification = 8,000×). Each ring-shaped bundle was weighed with a precision scale (MC210P, Sartorius, Gottingen, Germany) and reported as the mean and standard deviation of three measurements. The total length of each bundle was evaluated using ImageJ on high resolution photos of the specimens and reported as the mean and standard deviation of three measurements. To evaluate the porosity of the bundles, the volume fraction (ν) of each specimen was evaluated considering the following equation:

(1) $$\nu =\frac{\text{w}}{\text{L}\cdot \text{A}\cdot \rho }$$

where *w* is the weight of the specimen, *L* is length of the specimen, *A* is the cross-sectional area of the specimen, ρ is the density of the raw material (Nylon 6.6 = 1.14 g/cm^3^). The percentage of porosity for each sample category was evaluated considering the following equation (mean and standard deviation of *n* = 12 specimens for each category):

(2) Porosity=(1-ν)×100.

### Mechanical Characterization

The mechanical characterization of the bundles was conducted on a testing machine (Mod. 4465, Instron, Norwood, MA, United States) equipped with a ± 100 N load cell (Instron, Norwood, MA, United States). Twelve samples from each rotation speed were tested. The 2,500 rpm bundles had an average gauge length of 221.4 mm, the 1,750 rpm of 223.5 rpm while the 1,000 rpm bundles were 224.8 mm. The slightly different lengths of the bundles were caused by the prestretch of the fibers that was higher at higher rotational speeds. The tests consisted of a monotonic ramp to break (consistent with the ASTM D1414 Standard), in displacement control, where the crosshead speed was adjusted according to the sample length to reach a nominal strain rate of 0.33% s^–1^. Each specimen was immersed 2 min in PBS before the test. The ring shape of the bundles was exploited to load them between custom-made capstan grips to reduce the stress concentrations (see Supplementary Material) (Sensini et al., 2019a).

As a first step, the force-displacement data were converted to engineering stress-strain graphs based on the nominal cross-sectional area of the specimens. To compare the current samples to previously manufactured bundles and other tendon surrogates, the following mechanical properties were evaluated; yield stress (σ_*Y*_), yield strain (ε_*Y*_), elastic modulus (E), failure force (F_*F*_), failure stress (σ_*F*_), failure strain (ε_*F*_), unit work to yield (W_*Y*_), and unit work to failure (W_*F*_). The results of this analysis appear in the Supplementary Material section.

**FIGURE 3:**
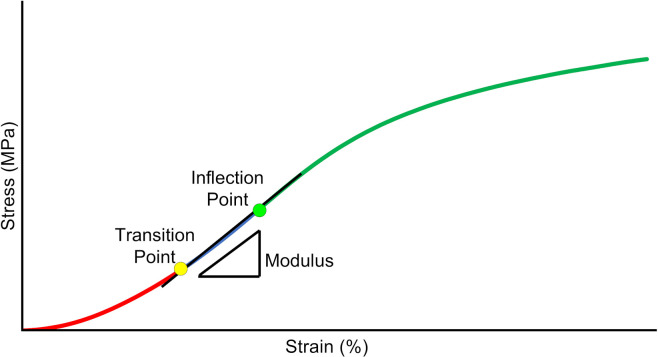
Schematic of critical data derived from the Tanaka et al. (2011) analysis of the low-strain portion of the stress-strain curve. Inflection point, is where the curve transitions from stiffening to softening. Transition point, is where the curve transitions from an exponential curve to a linear curve. Modulus, is the slope of the curve between the transition and inflection point.

To evaluate the biomechanically-relevant behavior in the low strain portion of the stress-strain response, the curves were analyzed to determine the inflection point of the curve (σ_*I*_), (ε_*I*_), the transition point from the toe region to the linear region (σ_*T*_), (ε_*T*_) and the slope in the linear region (E_*L*_). The inflection point is defined as the point where the stress-strain curve shifts from strain-stiffening to strain-softening. The transition point is where the toe region ends and the linear region begins. A MATLAB routine was developed for curve fitting and quantification of these parameters. As a first step, the *csaps* function was used to fit a cubic smoothing spline to the data. The smoothing parameter in the *csaps* routine was set to 0.7, to ensure smooth first and second derivative curves. The inflection point was then found by locating the zero point of the second derivative (Lee et al., 2017), and the inflection strain (ε_*I*_) and stress (σ_*I*_) were identified for that point (Figure 3).

Then, the σ-ε data below the inflection point was fit to a piecewise constitutive model to determine the transition point and linear region slope, using the technique of Tanaka et al. (2011). The model assumes an exponential fit below the transition point (stress, σ_*T*_, and strain, ε_*T*_) a linear modulus, E_*L*_, from the transition point to the inflection point and a continuous slope at the transition point as shown in Equation (3),

(3) $$\sigma =\left\{\begin{array}{c} \text{A}\left[\exp\left(\text{B}\varepsilon \right)-1\right],\forall \varepsilon \leq \varepsilon_{\text{T}}\\ {\text{E}}_{\text{L}}\left(\varepsilon -\varepsilon_{\text{T}}\right)+\sigma_{\text{T}},\forall \varepsilon >\varepsilon_{\text{T}} \end{array}\right\}$$

where *A* and *B* are empirical constants. Note that since the model requires a continuous slope at the transition point, the curve is fully characterized by selection of the parameters (A, B, and ε_*T*_). The *fmincon* function in MATLAB was used to optimize the parameters in the equation to minimize the mean square error between the model and the data.

### Statistical Analysis

To investigate the significance of the difference of distribution of diameters of the nanofibers between the 2,500, 1,750, and 1,000 rpm bundles (*n* = measures per sample category) a Kolmogorov–Smirnov test was used. The statistical significance of differences among the mechanical properties for the 2,500, 1,750, and 1,000 rpm bundles (*n* = 12 per sample category) were assessed with a one-way ANOVA test followed by a Tukey’s *post hoc*.

## Results

### Morphology of the Nanofibers and Bundles

**FIGURE 4:**
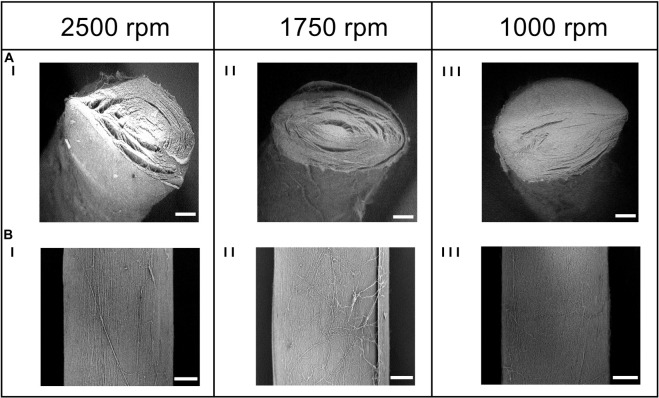
SEM investigation of the **(A)** cross-section (magnification = 200×; scale bar = 100 μm) and the **(B)** body (magnification = 260×; scale bar = 100 μm) of the electrospun bundles at: (I) 2,500 rpm, (II) 1,750 rpm and (III) 1,000 rpm.

**FIGURE 5:**
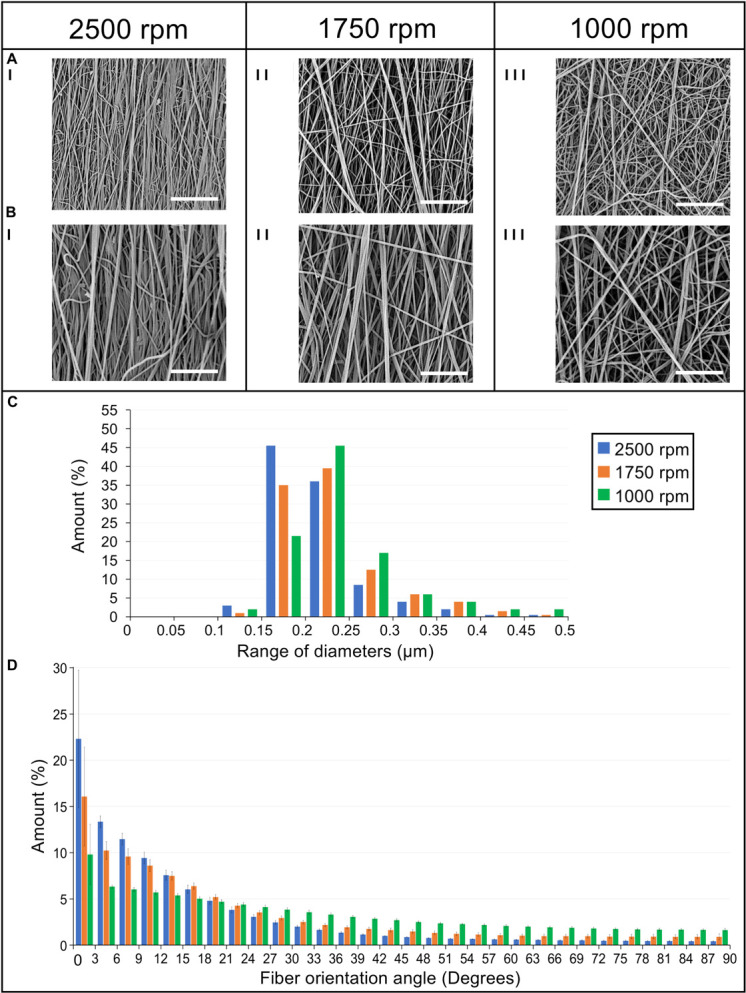
Morphological investigation of the bundles nanofibers. SEM images of the nanofibers at (I) 2,500 rpm, (II) 1,750 rpm, and (III) 1,000 rpm acquired at different magnifications: **(A)** 8,000× (scale bar = 10 μm); **(B)** 15,000× (scale bar = 5 μm). **(C)** Diameter distribution of the nanofibers in terms of percentage for the different ranges of diameters for the 2,500 rpm (blue bars), 1,750 rpm (orange bars), and 1,000 rpm (green bars). **(D)** Orientation analysis of the nanofibers in which an angle of 0° corresponds to the longitudinal axis of the specimen while an angle of 90° corresponds to the circumferential direction. The directionality histograms show the distribution in terms of percentage (mean and standard deviation) of nanofibers for the different ranges of angles for the 2,500 rpm (blue bars), 1,750 rpm (orange bars), and 1,000 rpm (green bars).

To mimic the tendon fibrils and fascicles (Kannus, 2000; Goh et al., 2014), electrospun bundles of nylon 6,6 nanofibers obtained with different peripheral speeds of the drum collector were compared. The SEM investigation (Figures 4, 5A,B) revealed that the bundle sections and the nanofibers were homogeneous, smooth, continuous, and with no defects such as beads.

The nanofibers of the 2,500 rpm bundles had a diameter of 0.21 ± 0.05 μm, the 1,750 rpm had a diameter of 0.23 ± 0.06 μm and the 1,000 rpm had a diameter of 0.24 ± 0.06 μm (see Figure 5C for the nanofibers diameter distribution). These are in the range of diameters for natural tendon fibrils (Kastelic et al., 1978; Kannus, 2000). The Kolmogorov–Smirnov test confirmed that the distributions of diameter of the nanofibers for the different samples families were different (*p* < 0.05). The mat thickness for the 2,500 rpm samples was 7 ± 1 μm, for the 1,750 rpm samples, 6 ± 1 μm and for the 1,000 rpm samples, 8 ± 2 μm. The diameter of the bundles for the 2,500 rpm was 613 ± 56 μm (length = 443 ± 4 mm), for the 1,750 rpm was 516 ± 24 μm (length = 447 ± 1 mm) while for the 1,000 rpm was 499 ± 35 μm (length = 450 ± 2 mm). These are in the range of diameters for natural tendon fascicles (Kastelic et al., 1978; Kannus, 2000; Goh et al., 2014). An increasing shrinkage of the nanofibers and bundles was noted passing from the 1,000 rpm to the 2,500 rpm. The weight of the bundles for the 2,500 rpm samples was 42.6 ± 3.77 mg (ν = 0.29 ± 0.04), for the 1,750 rpm samples, 27.2 ± 2.75 mg (ν = 0.3 ± 0.02) and for the 1,000 rpm samples, 20.7 ± 2.23 mg (ν = 0.2 ± 0.02). This resulted in a decreasing porosity (when the nanofibers anisotropy was increased). The porosity for the bundles at 1,000 rpm was 79.3 ± 1.56%, for the bundles at 1,750 rpm, 74.5 ± 1.74% and for the bundles at 2,500 rpm, 71.0 ± 4.04%.

### Orientation of the Bundles Nanofibers

The directional analysis carried out on the bundles showed a progressive decrease in the axial orientation of the nanofibers, passing from the 2,500 rpm to the 1,000 rpm, with a progressive increase in the circumferential scatter (Figure 5D). In fact, for the 2,500 rpm bundles the amount of nanofibers oriented in the range 0°–12° from the bundle’s axis was 57%, for the bundles spun at 1,750 rpm 45% of the nanofibers were in the range 0°–12°, while for the 1,000 rpm 28% were in the range 0°–12°. Conversely, the amount of nanofibers close to the circumferential direction, i.e. in the range 78°–90° from the bundle’s axis, was 2% for the 2,500 rpm bundles; for the bundles at 1,750 rpm 4% of the nanofibers were in the range 78°–90°, and for the bundles at 1,000 rpm 8% were in the range 78°–90°.

### Mechanical Properties of Bundles

Focusing on the early portion of the stress-strain curves, we applied the analysis described in section “Mechanical Characterization” to determine the transition point of the curves, the inflection point and the slope of the region between the two, referred to here as linear region modulus (E_*L*_). For a comparison with previously published studies, the “classical” parameters (stress, strain and work to yield and failure) are reported in the Supplementary Material.

**FIGURE 6:**
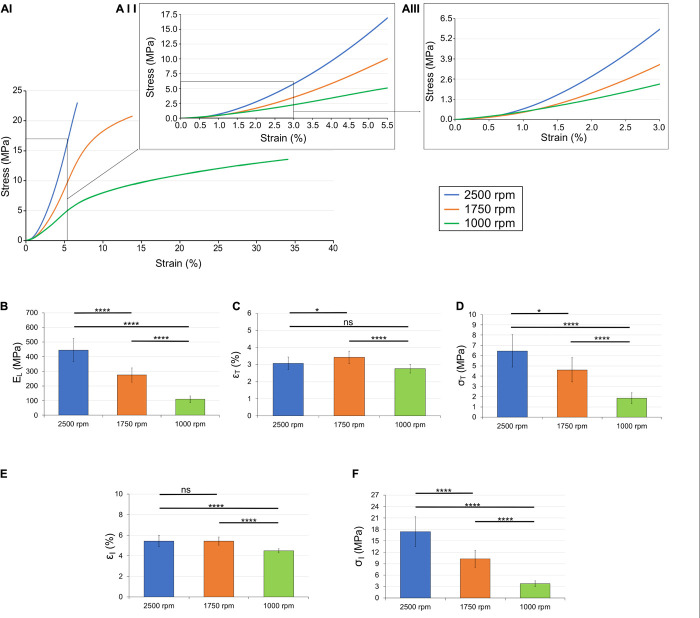
Mechanical behavior of the different bundles categories: **(AI)** overlay of the typical stress-strain curves; **(AII)** zoom-in up to the inflection points; **(AIII)** zoom-in up to the transition points. Comparison between the mechanical properties of the bundles: **(B)** linear region modulus; **(C)** transition strain; **(D)** transition stress; **(E)** inflection strain; and **(F)** inflection stress. The mean and standard deviation are represented for each group. The statistical significance of differences is indicated (*p ≤ 0.05, **p ≤ 0.01, ***p ≤ 0.001, ****p < 0.0001, and ns, not significant).

The results are shown in Figure 6 and the statistical analysis is shown in Table 1. The values for the modulus were all statistically different (1,000 rpm, 1.1 ± 0.23 MPa; 1,750 rpm, 2.7 ± 0.49 MPa; 2,500 rpm, 4.4 ± 0.78 MPa). The transition strain for the three sets of bundles were all around 3% (1,000 rpm, 2.8 ± 0.25%; 1,750 rpm, 3.4 ± 0.36%; 2,500 rpm, 3.1 ± 0.37%) with the only significant statistical difference being between the values for 1,000 and 1,750. However, the differences in transition stresses between groups were all statistically significant (1,000 rpm, 1.9 ± 0.53 MPa; 1,750 rpm, 4.6 ± 1.2 MPa; 2,500 rpm, 6.5 ± 1.6 MPa). The variations in inflection point showed similar behavior, with the inflection strain, being similar among the different groups, (1,000 rpm, 4.5 ± 0.19%; 1,750 rpm, 5.4 ± .40%; 2,500 rpm, 5.4 ± 0.54%) but the inflection stress, being quite different (1,000 rpm, 3.8 ± 0.79 MPa; 1,750 rpm, 10.3 ± 1.18 MPa; 2,500 rpm, 17.4 ± 3.9 MPa).

**TABLE 1:** Statistical significance of differences between the sample categories in terms of linear region modulus, transition strain, transition stress, inflection strain, and inflection stress.

	2,500 vs. 1,750 (rpm)	2,500 vs. 1,000 (rpm)	1,750 vs. 1,000 (rpm)
**ANOVA 1 (Tukey’s *post hoc*)**			
E_*L*_	**** (*P* < 0.0001)	**** (*P* < 0.0001)	**** (*P* < 0.0001)
ε_*T*_	* (*P* = 0.0330)	ns (*P* = 0.0684)	**** (*P* < 0.0001)
σ_*T*_	* (*P* = 0.0015)	**** (*P* < 0.0001)	**** (*P* < 0.0001)
ε_*I*_	Ns (*P* = 0.9997)	**** (*P* < 0.0001)	**** (*P* < 0.0001)
σ_*I*_	**** (*P* = 0.0001)	**** (*P* < 0.0001)	**** (*P* < 0.0001)

*p ≤ 0.05, **p ≤ 0.01, ***p ≤ 0.001, and ****p < 0.0001. ns, not significant.

The transition strains for 1,000 and 2,500 rpm were not significantly different (*p* = 0.0684) as well as the inflection strain between 1,750 and 2,500 rpm (*p* = 0.9997). All other values were significantly different between groups (Table 1).

## Discussion

Aiming to understand how the nanofibrous arrangement of electrospun nylon 6,6 prosthetic structures can be tuned to mimic the mechanical behavior of tendon fascicles at physiological levels of stress-strain, innovative nanofibrous bundles were produced with three different collector peripheral speeds.

Despite the different drum rotations, the nanofibers of all the bundles resulted in homogeneous, smooth, continuous fibers with the absence of defects such as beads. The mean cross-sectional diameter of the nanofibers was in the range of 0.21–0.24 μm (Figure 5C), in the same range as that of human tendon fibrils reported in literature (Kastelic et al., 1978; Kannus, 2000; Goh et al., 2014). The Kolmogorov–Smirnov test confirmed that the differences in the rotational speed contributed to the differences in the diameters of the nanofibers for the different sample families.

We have shown that by altering the processing parameters, electrospinning has the flexibility to create tendon surrogate materials with a range of material properties in the early, physiologically-important region of the stress-strain curve. It would be valuable to compare the properties of the nylon structures with those of natural tendon tissue. However, the variability of testing protocols in the literature makes this difficult. Perhaps more importantly, few papers report the parameters evaluated in the current study (i.e., inflection point, transition point, and modulus of the portion in between). But there are a few studies that focus on these parameters in rat tendon. Lee et al. (2017) studied the effects of damage on the inflection point, transition point, and modulus of rat tail tendon and later Lee and Elliott (2019) looked at the same parameters for rat plantaris tendon. These tests were conducted under a different protocol that the stress-strain tests in the current study. Most notably, Lee et al. (2017) preconditioned the tendon sample before testing, while we did not. In addition, the strain rates used were three times higher (1% s^–1^ for vs. 0.33% s^–1^). Even though these tests followed a different protocol than the testing done in the current study, it is interesting to compare the numerical values for the listed parameters.

Lee et al. (2017) reported transition strain and stress for undamaged, rat tail tendon fascicles in the range of 1.5–1.7% and 4.9–5.3 MPa, respectively. For the inflection strain and stress, they reported 2.2–2.4% and 12–16 MPa, respectively. While the stress values are in the ranges reported in the results section for the nylon structures, the strain values are lower by a factor of two. In addition, the modulus for the undamaged rat tail tendon fascicles was in the range 870–980 MPa, which is a factor of two higher than the highest average modulus of the nylon samples reported in the results. Interestingly, the modulus reported in Lee and Elliott (2019) for rat plantaris tendon is in the range of 250–335 MPa, similar to the nylon, but the transition and inflection points are all much higher (*p* = 9.5–10%; *q* = 9 − 13 MPa; ε_*I*_ = 15–16%; σ_*I*_ = 25–34 MPa).

The orientation analysis of the nanofibers showed that increasing the speed of the collector, for each increment, 1,000 to 1,750 to 2,500 rpm, a similar increase of the fraction of nanofibers in the range 0°–3° (i.e., axially aligned) of about 6%. For the same conditions, in the range of 87°–90° (i.e., nearly circumferential) a progressive decrease of about 0.6% was noted. The higher degree of alignment noted for the 2,500 rpm bundles (range 0°–12° of 57%) showed a similar arrangement to natural tendon collagen fibrils reported in literature (Provenzano and Vanderby, 2006; Sivaguru et al., 2010; Rouède et al., 2017). The progressive axial orientation of the nanofibers inside the bundles, also caused a slight shrinkage of the nanofibers, reducing the overall length of the bundles from the nominal drum circumference by approximately the 4.5% for the 1,000 rpm, 5.1% for the 1,750 rpm and 6% for the 2,500 rpm. The reduction of the nanofiber shrinkage and the increase in the porosity of the bundles, passing from the 2,500 rpm to the 1,000 rpm, also caused a reduction of the related volume fraction of the bundles of approximately 13.8 %, from the 2,500 rpm to the 1,750 rpm, and an additional 24.1% from the 1,750 rpm to the 1,000 rpm. These data were consistent with the previous literature (Sensini et al., 2019a).

To reach a faithful biomimicry of the natural tissues, a bioinspired morphology has to guarantee adequate biomechanical properties. The main advantage of using electrospun bundles of aligned nanofibers, is their non-linear mechanical behavior, similar to tendon (Goh et al., 2014; Murphy et al., 2016; Sensini and Cristofolini, 2018). This biomimetic mix between structure and mechanics makes this electrospun structure particularly suitable to mimic the tendon fascicles (Bosworth et al., 2014; Pauly et al., 2016; Laranjeira et al., 2017; Sensini and Cristofolini, 2018; Sensini et al., 2019a). All the different bundles showed a well-defined toe region, caused by the progressive recovery from the crimped state of the low friction nylon 6,6 nanofibers after the removal from the drum (Gotti et al., 2020). The bundles also showed a progressive shift from a brittle (at 2,500 rpm bundles) to an increasingly ductile behavior (at 1,750 and 1,000 rpm) (Figure 6 and Supplementary Material). This phenomenon caused a statistically significant reduction of the failure force from the 2,500 rpm to the 1,750 rpm of 35.9% (*P* < 0.0001) and of 61.7% (*P* < 0.0001) from the 2,500 rpm to the 1,000 rpm. The force loss caused a small reduction (not significant) in the failure stresses between the 2,500 rpm σ_*F*_ = 23.9 ± 5.94 MPa and the 1,750 rpm σ_*F*_ = 21.5 ± 2.35 MPa (*P* = 0.3046) and a significant reduction at 1,000 rpm σ = 13.7 ± 2.33 MPa (*P* < 0.0001) (see Supplementary Material). However, all these stress values are in the range of human tendon fascicles such as the iliopsoas (σ_*F*_ = 4.7–29.8 MPa) or the Achilles (σ_*F*_ = 12–37.9 MPa) tendons (Hanson et al., 2012). Concerning the failure strains, the progressive loss of anisotropy of the nanofibers, due to the reduced drum speed, caused statistically significant increases of their values between the 2,500 rpm ε_*F*_ = 6.73 ± 0.73%, the 1,750 rpm ε_*F*_ = 13.8 ± 3.68% (105% increment; *P* = 0.0006) and the 1,000 rpm ε_*F*_ = 31.9 ± 6.15% (374% increment; *P* < 0.0001). The failure strains of the different bundles were in the wide range of those reported in literature ranging between human patellar tendon (ε_*F*_ = 5–60%), iliopsoas (ε_*F*_ = 14.8–24.9%) and Achilles (ε_*F*_ = 9.4–19.8%) (Butler et al., 1986; Hanson et al., 2012; Goh et al., 2014; Murphy et al., 2016). The elastic modulus of the bundles showed a statistically significant decrease (*P* < 0.0001) between the axial orientation and the more randomic arrangement of the nanofibers (Figure 6). In particular for the 2,500 rpm case, the elastic modulus was *E* = 486 ± 94 MPa, for the 1,750 rpm, it was *E* = 288 ± 47 MPa (decrement of 40.7%) and for the 1,000 rpm and it was *E* = 113 ± 25 MPa (decrement of 76.7%). These values resulted in the same range as of the human tendon fascicle of the iliopsoas (*E* = 39.9–232.6 MPa), Achilles (*E* = 138.2 ± 426 MPa) (Hanson et al., 2012). These results were in line with the previous studies carried out on nylon 6,6 electrospun materials (Yao et al., 2014; Sensini and Cristofolini, 2018, Sensini et al., 2019a).

## Conclusion

From the manufacturing point of view, we have shown that we can mimic the physiologically-relevant early-strain portion of natural tendon fascicle stress-strain behavior through electrospinning. In fact, we were able to obtain similar transition and inflection stresses and strains, as well as linear-region modulus. By matching several of these nylon 6,6 bundles together it will be also possible to increase the multiscale complexity of the overall structure, obtaining hierarchical devices suitable to mimic the whole structure and mechanics of tendon (Sensini et al., 2018a, 2020, 2019a,b). The same approach can be used to tune the manufacturing parameters using other materials, such as PLA and collagen, to obtain resorbable scaffolds and prosthetic devices.

## Data Availability Statement

The raw data supporting the conclusions of this article will be made available by the authors, without undue reservation.

## Author Contributions

MS, LC, and AS conceptualized the study and reviewed and edited the draft. AS wrote the original draft and prepared the figures with help from EE. AS produced the electrospun specimens and performed the morphological and mechanical characterizations. AS and AZ optimized the electrospinning procedure to obtain bundles. AS with the help of CG, elaborated the mechanical data. EE, EB, and MS performed the mechanical modeling of the inflection and transition points and analyzed these data. AZ and LC supervised the work and were responsible for the funding acquisition. All authors listed have made a substantial, direct and intellectual contribution to the work, and approved it for publication.

## Conflict of Interest

AS, LC, and AZ hold an international patent on a related invention (“Hierarchical multiscale electrospun scaffold for the regeneration and/or replacement of the tendinous/ligamentous tissue and a method for its production” WO2018/229615 A1 of 20 December 2018). The remaining authors declare that the research was conducted in the absence of any commercial or financial relationships that could be construed as a potential conflict of interest.

## References

[B1] Bosworth L. A. Alam N. Wong J. K. Downes S. (2013). Investigation of 2D and 3D electrospun scaffolds intended for tendon repair. *J. Mater. Sci. Mater. Med.* 24 1605–1614. 10.1007/s10856-013-4911-8 23504088

[B2] Bosworth L. A. Downes S. (eds) (2011). *Electrospinning for Tissue Regeneration*, 1st Edn. Cambridge: Woodhead Publishing, 10.1016/B978-1-84569-741-9.50001-X

[B3] Bosworth L. A. Rathbone S. R. Bradley R. S. Cartmell S. H. (2014). Dynamic loading of electrospun yarns guides mesenchymal stem cells towards a tendon lineage. *J. Mech. Behav. Biomed. Mater.* 39 175–183. 10.1016/j.jmbbm.2014.07.009 25129861 PMC4180006

[B4] Butler D. L. Kay M. D. Stouffer D. C. (1986). Comparison of material properties in fascicle-bone units from human patellar tendon and knee ligaments. *J. Biomech.* 19 425–432. 10.1016/0021-9290(86)90019-9 3745219

[B5] Chen J. Xu J. Wang A. Zheng M. (2014). Scaffolds for tendon and ligament repair: review of the efficacy of commercial products. *Expert Rev. Med. Devices* 6 61–73. 10.1586/17434440.6.1.61 19105781

[B6] Domingues R. M. A. Chiera S. Gershovich P. Motta A. Reis R. L. Gomes M. E. (2016). Enhancing the biomechanical performance of anisotropic nanofibrous scaffolds in tendon tissue engineering: reinforcement with cellulose nanocrystals. *Adv. Healthc. Mater.* 5 1364–1375. 10.1002/adhm.201501048 27059281

[B7] Goh K. L. Listrat A. Béchet D. (2014). Hierarchical mechanics of connective tissues: integrating insights from nano to macroscopic studies. *J. Biomed. Nanotechnol.* 10 2464–2507. 10.1166/jbn.2014.1960 25992406

[B8] Gomes M. E. Reis R. L. Rodrigues M. T. (2015). *Tendon Regeneration: Understanding Tissue Physiology and Development to Engineer Functional Substitutes.* London: Academic Press.

[B9] Gotti C. Sensini A. Fornaia G. Gualandi C. Zucchelli A. Focarete M. L. (2020). Biomimetic hierarchically arranged nanofibrous structures resembling the architecture and the passive mechanical properties of skeletal muscles?: a step forward toward artificial muscle. *Front. Bioeng. Biotechnol.* 8:767. 10.3389/fbioe.2020.00767 32766220 PMC7379046

[B10] Han W. M. Heo S. J. Driscoll T. P. Smith L. J. Mauck R. L. Elliott D. M. (2013). Macro- to microscale strain transfer in fibrous tissues is heterogeneous and tissue-specific. *Biophys. J.* 105 807–817. 10.1016/j.bpj.2013.06.023 23931328 PMC3736685

[B11] Hansen K. A. Weiss J. A. Barton J. K. (2002). Recruitment of tendon crimp with applied tensile strain. *J. Biomech. Eng.* 124 72–77. 10.1115/1.1427698 11871607

[B12] Hanson P. Aagaard P. Magnusson S. P. (2012). Biomechanical properties of isolated fascicles of the iliopsoas and achilles tendons in African American and Caucasian men. *Ann. Anat.* 194 457–460. 10.1016/j.aanat.2012.03.007 22583513

[B13] Kannus P. (2000). Structure of the tendon connective tissue. *Scand. J. Med. Sci. Sports* 10 312–320. 10.1034/j.1600-0838.2000.010006312.x 11085557

[B14] Kastelic J. Galeski A. Baer E. (1978). The multicomposite structure of tendon. *Connect. Tissue Res.* 6 11–23. 10.3109/03008207809152283 149646

[B15] Krysiak Z. J. Gawlik M. Z. Knapczyk-Korczak J. Kaniuk L. Stachewicz U. (2020). Hierarchical composite meshes of electrospun PS microfibers with PA6 nanofibers for regenerative medicine. *Materials (Basel)* 13 11–13. 10.3390/MA13081974 32340243 PMC7216289

[B16] Lake S. P. Miller K. S. Elliott D. M. Soslowsky L. J. (2009). Effect of fiber distribution and realignment on the nonlinear and inhomogeneous mechanical properties of human supraspinatus tendon under longitudinal tensile loading. *J. Orthop. Res.* 27 1596–1602. 10.1002/jor.20938 19544524 PMC2813200

[B17] Laranjeira M. Domingues R. M. A. Costa-Almeida R. Reis R. L. Gomes M. E. (2017). 3D mimicry of native-tissue-fiber architecture guides tendon-derived cells and adipose stem cells into artificial tendon constructs. *Small* 13 1–13. 10.1002/smll.201700689 28631375

[B18] Lee A. H. Elliott D. M. (2019). Multi-scale loading and damage mechanisms of plantaris and rat tail tendons. *J. Orthop. Res.* 37 1827–1837. 10.1002/jor.24309 30977538 PMC6790141

[B19] Lee A. H. Szczesny S. E. Santare M. H. Elliott D. M. (2017). Investigating mechanisms of tendon damage by measuring multi-scale recovery following tensile loading. *Acta Biomater.* 57 363–372. 10.1016/j.actbio.2017.04.011 28435080 PMC6688648

[B20] Liu Z. (1991). Scale space approach to directional analysis of images. *Appl. Opt.* 30 1369–1373. 10.1364/AO.30.001369 20700292

[B21] Lynch H. A. Johannessen W. Wu J. P. Jawa A. Elliott D. M. (2003). Effect of fiber orientation and strain rate on the nonlinear uniaxial tensile material properties of tendon. *J. Biomech. Eng.* 125 726–731. 10.1115/1.1614819 14618932

[B22] Maitz M. F. (2015). Applications of synthetic polymers in clinical medicine. *Biosurf. Biotribol.* 1 161–176. 10.1016/j.bsbt.2015.08.002

[B23] Murphy W. Black J. Hastings G. (eds) (2016). *Handbook of Biomaterial Properties*, 2nd Edn. Hastings: Springer, 10.1007/978-1-4939-3305-1

[B24] O’Connor R. A. McGuinness G. B. (2016). Electrospun nanofibre bundles and yarns for tissue engineering applications: a review. *Proc. Inst. Mech. Eng. H J. Eng. Med.* 230 987–998. 10.1177/0954411916656664 28095765

[B25] Pauly H. M. Kelly D. J. Popat K. C. Trujillo N. A. Dunne N. J. McCarthy H. O. (2016). Mechanical properties and cellular response of novel electrospun nanofibers for ligament tissue engineering: effects of orientation and geometry. *J. Mech. Behav. Biomed. Mater.* 61 258–270. 10.1016/j.jmbbm.2016.03.022 27082129

[B26] Pauly H. M. Sathy B. N. Olvera D. McCarthy H. O. Kelly D. J. Popat K. C. (2017). Hierarchically structured electrospun scaffolds with chemically conjugated growth factor for ligament tissue engineering. *Tissue Eng. A* 23 823–836. 10.1089/ten.tea.2016.0480 28350237

[B27] Provenzano P. P. Vanderby R. (2006). Collagen fibril morphology and organization: Implications for force transmission in ligament and tendon. *Matrix Biol.* 25 71–84. 10.1016/j.matbio.2005.09.005 16271455

[B28] Ramakrishna S. Fujihara K. Teo W. E. Lim T. C. Ma Z. (2005). *An Introduction to Electrospinning and Nanofibers.* London: World Scientific Publishing. 10.1142/5894

[B29] Rouède D. Schaub E. Bellanger J. J. Ezan F. Scimeca J. C. Baffet G. (2017). Determination of extracellular matrix collagen fibril architectures and pathological remodeling by polarization dependent second harmonic microscopy. *Sci. Rep.* 7:12197. 10.1038/s41598-017-12398-0 28939903 PMC5610346

[B30] Schindelin J. Arganda-Carreras I. Frise E. Kaynig V. Longair M. Pietzsch T. (2012). Fiji: an open-source platform for biological-image analysis. *Nat. Methods* 9 676–682. 10.1038/nmeth.2019 22743772 PMC3855844

[B31] Schneider C. A. Rasband W. S. Eliceiri K. W. (2012). NIH Image to ImageJ: 25 years of image analysis. *Nat. Methods* 9 671–675. 10.1038/nmeth.2089 22930834 PMC5554542

[B32] Sensini A. Cristofolini L. (2018). Biofabrication of electrospun scaffolds for the regeneration of tendons and ligaments. *Materials (Basel)* 11:1963. 10.3390/ma11101963 30322082 PMC6213815

[B33] Sensini A. Cristofolini L. Focarete M. L. Belcari J. Zucchelli A. Kao A. (2018a). High-resolution x-ray tomographic morphological characterisation of electrospun nanofibrous bundles for tendon and ligament regeneration and replacement. *J. Microsc.* 272 196–206. 10.1111/jmi.12720 29797707

[B34] Sensini A. Cristofolini L. Gualandi C. Focarete M. L. Belcari J. Zucchelli A. (2018b). *Hierarchical Multiscale Electrospun Scaffold for the Regeneration and/or Replacement of the Tendinous/Ligamentous Tissue and a Method for its Production.* WO2018/229615A1.

[B35] Sensini A. Cristofolini L. Zucchelli A. Focarete M. L. Gualandi C. de Mori A. (2020). Hierarchical electrospun tendon-ligament bioinspired scaffolds induce changes in fibroblasts morphology under static and dynamic conditions. *J. Microsc.* 277 160–169. 10.1111/jmi.12827 31339556

[B36] Sensini A. Gotti C. Belcari J. Zucchelli A. Focarete M. L. Gualandi C. (2019a). Morphologically bioinspired hierarchical Nylon 6.6 electrospun assembly recreating the structure and performance of tendons and ligaments. *Med. Eng. Phys.* 71 79–90. 10.1016/j.medengphy.2019.06.019 31262555

[B37] Sensini A. Gualandi C. Cristofolini L. Tozzi G. Dicarlo M. Teti G. (2017). Biofabrication of bundles of poly(lactic acid)-collagen blends mimicking the fascicles of the human Achille tendon. *Biofabrication* 9:015025. 10.1088/1758-5090/aa6204 28224971

[B38] Sensini A. Gualandi C. Focarete M. L. Belcari J. Zucchelli A. Boyle L. (2019b). Multiscale hierarchical bioresorbable scaffolds for the regeneration of tendons and ligaments. *Biofabrication* 11:35026. 10.1088/1758-5090/ab20ad 31071692

[B39] Sensini A. Gualandi C. Zucchelli A. Boyle L. Kao A. P. Reilly G. C. (2018c). Tendon Fascicle-Inspired Nanofibrous Scaffold of Polylactic acid/Collagen with Enhanced 3D-Structure and Biomechanical Properties. *Sci. Rep.* 8:17167. 10.1038/s41598-018-35536-8 30464300 PMC6249227

[B40] Sensini A. Massafra G. Gotti C. Zucchelli A. Cristofolini L. (2021). Tissue engineering for the insertions of tendons and ligaments: an overview of electrospun biomaterials and structures. *Front. Bioeng. Biotechnol.* 9:98. 10.3389/fbioe.2021.645544 33738279 PMC7961092

[B41] Silver F. H. Christiansen D. L. Snowhill P. B. Chen Y. (2000). Role of storage on changes in the mechanical properties of tendon and self-assembled collagen fibers. *Connect. Tissue Res.* 41 155–164. 10.3109/03008200009067667 10992161

[B42] Sivaguru M. Durgam S. Ambekar R. Luedtke D. Fried G. Stewart A. (2010). Quantitative analysis of collagen fiber organization in injured tendons using Fourier transform-second harmonic generation imaging. *Opt. Express* 18 24983–24993. 10.1364/oe.18.024983 21164843

[B43] Tanaka M. L. Weisenbach C. A. Miller M. C. Kuxhaus L. (2011). A continuous method to compute model parameters for soft biological materials. *J. Biomech. Eng-ASME.* 133:074502. 10.1115/1.4004412 21823751

[B44] Weiss J. A. Gardiner J. C. Bonifasi-Lista C. (2002). Ligament material behavior is nonlinear, viscoelastic and rate-independent under shear loading. *J. Biomech.* 35 943–950. 10.1016/S0021-9290(02)00041-6 12052396

[B45] Wren T. A. Yerby S. A. Beaupré G. S. Carter D. R. (2001). Mechanical properties of the human achilles tendon. *Clin. Biomech.* 16 245–51. 10.1016/s0268-0033(00)00089-9 11240060

[B46] Yao J. Bastiaansen C. W. M. Peijs T. (2014). High strength and high modulus electrospun nanofibers. *Fibers* 2 158–187. 10.3390/fib2020158

