# Supplementary material for: Tuning the Structure of Nylon 6,6 Electrospun Bundles to Mimic the Mechanical Performance of Tendon Fascicles
Source: Front Bioeng Biotechnol. 2021 Apr 6;9:626433. doi: 10.3389/fbioe.2021.626433 (PMC8056020; doi:10.3389/fbioe.2021.626433)
Supplement: Supplementary file 1 [file Data_Sheet_1.docx]

Supplementary Material

# The aim of this Supplementary Material is to provide additional mechanical data, to enable comparisons with previously published similar works. While the main paper focuses on the low-strain region of the stress-strain curve, specifically on the Transition and Inflection points and on the Elastic modulus between those two points, here the more classical parameters (yield and failure and average modulus from transition to failure) are reported.

# 1. Classical uniaxial stress-strain characterization procedure

The ring-shaped bundles were loaded between two dedicated custom-made capstan grips to reduce the stress concentrations (Figure S1 A). The force-displacement curves were converted to stress-strain graphs by dividing the force by the nominal cross-sectional area of the specimen.

The following indicators were considered, (Figure S1 B and C): Yield Stress (σ_Y_), Yield Strain (ε_Y_), Elastic Modulus from transition to failure (E), Failure Force (F_F_), Failure Stress (σ_F_), Failure Strain (ε_F_), Unit Work to Yield (W_Y_), Unit Work to Failure (W_F_).


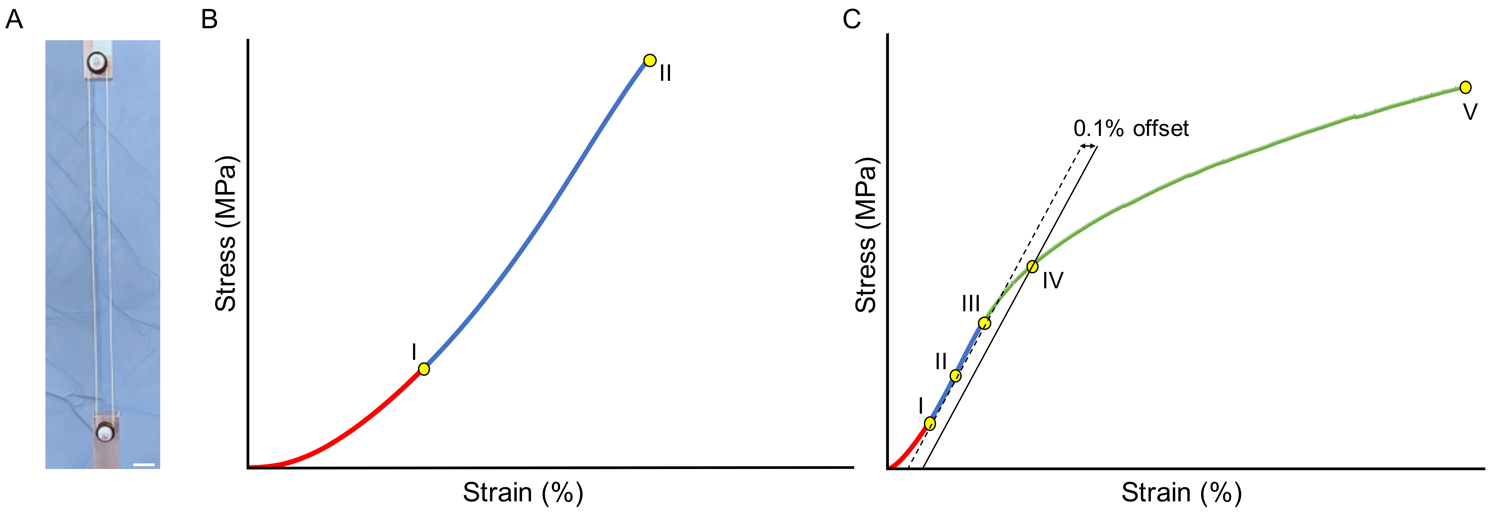


**FIGURE S1|** Mechanical characterization setup and data post processing. (A) Capstan grips for testing the bundles (scale bar = 10 mm). The bundles produced at 2500 rpm (B) showed a brittle behavior and those produced at 1750 rpm and 1000 rpm (C) a more ductile one, requiring slightly different approaches for the data analysis. In this analysis, the initial toe region, from 0 MPa up to the 25% (BI and CI) of the failure stress (BII and CV), was disregarded. (B) Typical stress-strain curve of a 2500 rpm bundle. The elastic modulus was calculated as the slope of the linear regression between (BI) and the failure stress (BII). (C) Typical stress-strain curve for a 1000 rpm bundle (valid also for the 1750 rpm ones). A first-guess yield stress was identified (CIII) and then the 50% point between (CI) and (CIII) was calculated (CII). A first linear regression (dashed line) was applied from (CI) and (CII). A second line parallel to the first regression was drawn with an offset of 0.1% strain (solid line). The limit of proportionality (σ_Y_) was defined as the intersection between the solid line and the stress-strain curve (CIV). The elastic modulus was calculated as the slope of a new regression line between (CI) and (CIV). The failure stress (σ_F_) was identified as the highest stress in the entire curve (CV). The unit work to yield (W_Y_) and to failure (W_F_) were calculated as integrals of the area under the curves (with the trapezoids method).

# Statistical analysis

The significance of differences between the mechanical properties for the 2500 rpm, 1750 rpm and 1000 rpm bundles (n = 12 per sample category) was assessed with a one-way ANOVA test followed by a Tukey post-hoc. The significance of differences between the mechanical properties for the 1750 rpm and 1000 rpm bundles (n = 12 per sample category) in terms of yield stress, yield strain and work to yield were assessed with an unpaired T-test with a Welch’s correction for inequality of variances.

# 3. Results of the classical mechanical characterization

**
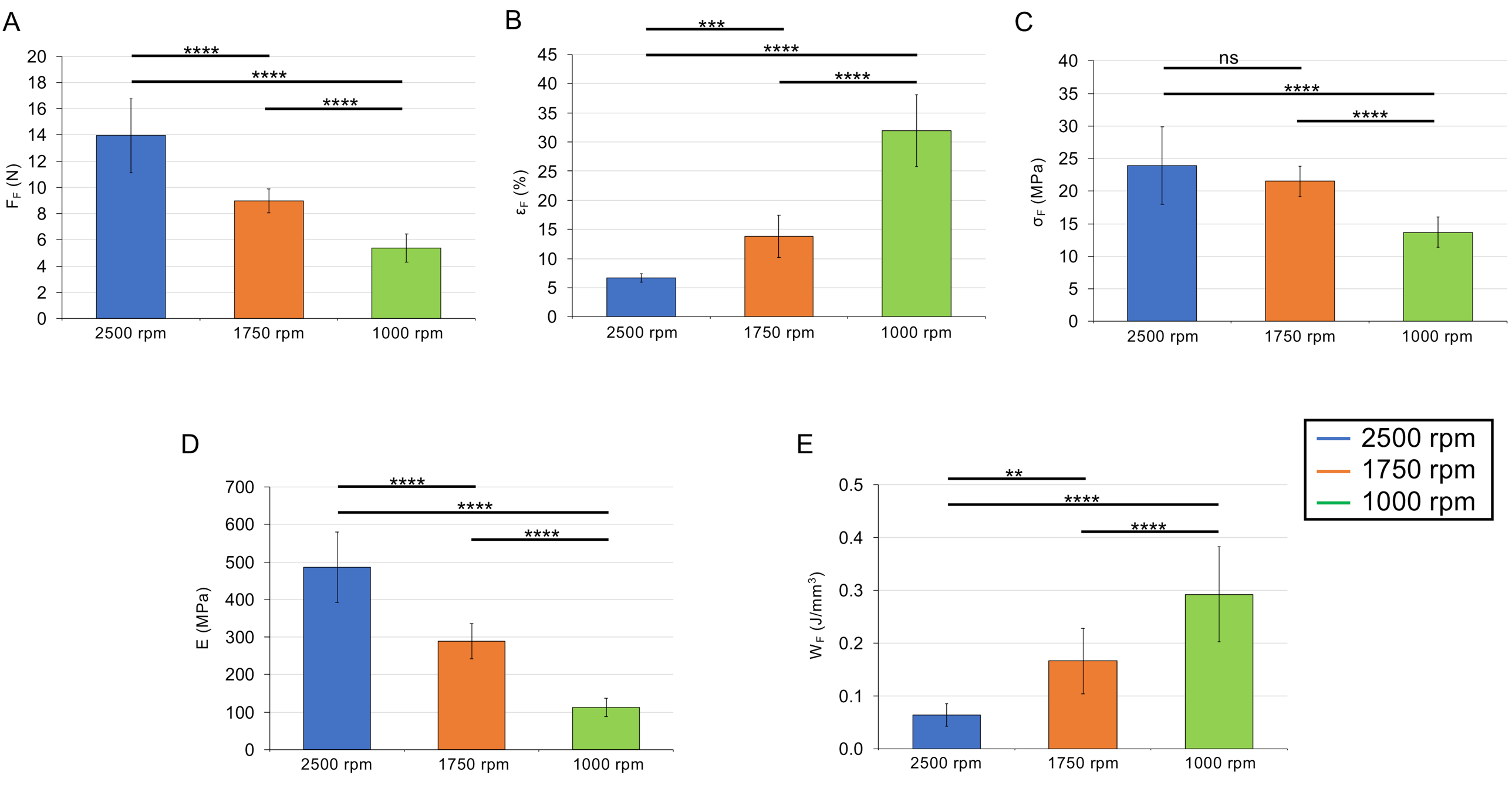
**

**FIGURE S2|** Classical uniaxial tensile properties of bundles (2500 rpm = blue; 1750 = orange; 1000 rpm = green). (A) failure force; (B) failure strain; (C) failure stress; (D) elastic modulus; (E) work to failure. The statistical significance of differences is indicated (*p ≤ 0.05, **p ≤ 0.01, ***p ≤ 0.001, ****p <0.0001, ns = not significant).

**TABLE S1|** Summary of the classical uniaxial tensile properties of the bundles (mean and standard deviation over the n = 12 specimens of each group).

|  | | **2500**  **(rpm)** | | **1750**  **(rpm)** | | | **1000**  **(rpm)** |  |
| --- | --- | --- | --- | --- | --- | --- | --- | --- |
| F_F_ | (N) | | 14.0 ± 2.83 | | 8.97 ± 0.91 | 5.37 ± 1.09 | | |
| σ_Y_ | (MPa) | | - | | 16.3 ± 4.00 | 5.57 ± 1.06 | | |
| σ_F_ | (MPa) | | 23.9 ± 5.94 | | 21.5 ± 2.35 | 13.7 ± 2.33 | | |
| ε_Y_ | (%) | | - | | 7.67 ± 0.79 | 6.13 ± 0.20 | | |
| ε_F_ | (%) | | 6.73 ± 0.73 | | 13.8 ± 3.68 | 31.9 ± 1.09 | | |
| E | (MPa) | | 486 ± 94 | | 288 ± 47 | 113 ± 25 | | |
| W_Y_ | (J/mm^3^) | | - | | 0.05 ± 0.02 | 0.01 ± 0.003 | | |
| W_F_ | (J/mm^3^) | | 0.06 ± 0.02 | | 0.17 ± 0.06 | 0.29 ± 0.09 | | |

**TABLE S2|** Statistical significance of differences between the sample categories (*p ≤ 0.05, **p ≤ 0.01, ***p ≤ 0.001, ****p <0.0001, ns = not significant)

|  | | **2500 vs 1750**  **(rpm)** | | **2500 vs 1000 (rpm)** | **1750 vs 1000**  **(rpm)** |  |
| --- | --- | --- | --- | --- | --- | --- |
| ANOVA 1  (Tukey post-hoc) | |  | |  |  | |
| F_F_ | (N) | | ****  (P < 0.0001) | ****  (P < 0.0001) | ****  (P < 0.0001) | |
| σ_F_ | (MPa) | | ns  (P = 0.3046) | ****  (P < 0.0001) | ****  (P < 0.0001) | |
| ε_F_ | (%) | | ***  (P = 0.0006) | ****  (P < 0.0001) | ****  (P < 0.0001) | |
| E | (MPa) | | ****  (P < 0.0001) | ****  (P < 0.0001) | ****  (P < 0.0001) | |
| W_F_ | (J/mm^3^) | | **  (P = 0.0016) | ****  (P < 0.0001) | ****  (P < 0.0001) | |
| Unpaired T-test  (Welch’s correction) | | | | | | |
| σ_Y_ | (MPa) | | - | - | ****  (P < 0.0001) | |
| ε_Y_ | (%) | | - | - | ***  (P = 0.0001) | |
| W_Y_ | (J/mm^3^) | | - | - | ****  (P < 0.0001) | |
